# A face for all seasons: Searching for context-specific leadership traits and discovering a general preference for perceived health

**DOI:** 10.3389/fnhum.2014.00792

**Published:** 2014-11-05

**Authors:** Brian R. Spisak, Nancy M. Blaker, Carmen E. Lefevre, Fhionna R. Moore, Kleis F. B. Krebbers

**Affiliations:** ^1^Department of Management and Organization, VU University AmsterdamAmsterdam, Netherlands; ^2^Department of Social and Organizational Psychology, VU University AmsterdamAmsterdam, Netherlands; ^3^Centre for Decision Research, Leeds University Business School, University of LeedsLeeds, UK; ^4^School of Psychology, University of DundeeDundee, UK

**Keywords:** leadership, prototypes, contingency, categorization, face perception, attractiveness, health, intelligence

## Abstract

Previous research indicates that followers tend to contingently match particular leader qualities to evolutionarily consistent situations requiring collective action (i.e., context-specific cognitive leadership prototypes) and information processing undergoes categorization which ranks certain qualities as first-order context-general and others as second-order context-specific. To further investigate this contingent categorization phenomenon we examined the “attractiveness halo”—a first-order facial cue which significantly biases leadership preferences. While controlling for facial attractiveness, we independently manipulated the underlying facial cues of health and intelligence and then primed participants with four distinct organizational dynamics requiring leadership (i.e., competition vs. cooperation between groups and exploratory change vs. stable exploitation). It was expected that the differing requirements of the four dynamics would contingently select for relatively healthier- or intelligent-looking leaders. We found perceived facial intelligence to be a second-order context-specific trait—for instance, in times requiring a leader to address between-group cooperation—whereas perceived health is significantly preferred across all contexts (i.e., a first-order trait). The results also indicate that facial health positively affects perceived masculinity while facial intelligence negatively affects perceived masculinity, which may partially explain leader choice in some of the environmental contexts. The limitations and a number of implications regarding leadership biases are discussed.

## Introduction

Investigating evolved cognitive mechanisms mediating the connection between environmental triggers and leadership emergence is a burgeoning field that works to add a biologically inspired expansion to traditional models of contingent and implicit leadership (e.g., Fiedler, 1964; Lord et al., 1982; Spisak et al., 2012). Such research helps to clarify leadership biases and their potential impact on everything from voting behavior and CEO succession outcomes to informal leadership emergence in local networks. The underlying psychological mechanisms facilitating this emergence have been referred to as context-specific cognitive leadership prototypes (Spisak et al., 2011).

Such psychological adaptations are arguably part of the human evolutionary trajectory toward increasingly complex social group strategies as a means to maintain and increase fitness in competitive environments (e.g., Couzin et al., 2005). As groups grow in size and complexity, costly risks arise in the form of reoccurring coordination problems which select for adaptive solutions—including leadership (Van Vugt et al., 2008). Indeed, leadership has been observed across cultures (Brown, 1991) and emerges with minimal conscious effort (De Cremer and Van Vugt, 2002). Collective action challenges benefiting from such a social adaptation includes the successful management of competition and cooperation between groups. Poor coordination during competition can lead to failure in the presence of a *raiding* out-group whereas the ability to effectively cooperate between groups can, in *trading* situations, reduce the costs of conflict and increase success. Further, research on modern organizational behavior has demonstrated that management efforts to correctly orient a team either toward competition or cooperation depending on the task can have a significant impact on performance (Beersma et al., 2003). Thus, these “raiding vs. trading” dynamics were (and are) powerful forces in the adaptive landscape of group behavior (e.g., Wrangham and Peterson, 1996; Bowles, 2009; Van Vugt, 2009).

There is also the need to effectively divide the investment of time and energy between finding new resources vs. extracting rewards from existing resources—known as the “Exploration-Exploitation Dilemma” in the organizational science literature (March, 1991) and related to ecological theories such as “Optimal Foraging Theory” (MacArthur and Pianka, 1966). A balance needs to be made where a group must not over-exploit for fear of becoming obsolete relative to more exploratory groups. On the other hand, a group must work to competitively capitalize on an established resource before shifting to more exploratory alternatives. Effectively managing the exploration-exploitation dilemma subsequently increases (or decreases) group success—be it migratory decisions about food or executive strategies in free markets. As with raiding vs. trading, the pressures of exploration vs. exploitation appear to have also had an impact on human evolution. Specific neural mechanisms, occupying distinct substrates, exist for processing information regarding this dilemma (e.g., Daw et al., 2006). Cohen et al. (2007), for instance, report that this neuromodularity reacts to estimates of uncertainty and expected utility (i.e., fundamental aspects of the exploration-exploitation dilemma). Relatedly, McDermott et al. (2008) connects this underlying evolved logic of optimal foraging to the well-established decision-making assumptions of prospect theory (Kahneman and Tversky, 1979). Such evidence points to cognitive systems which have been selected for to solve reoccurring problems associated with exploring new alternatives vs. exploiting an established option.

It is argued that (1) leadership (i.e., the ability to influence others and act as a focal point of coordinated behavior to achieve group objectives; e.g., Yukl, 2006) is an adaptation to manage challenges associated with exploration vs. exploitation and competition vs. cooperation and that (2) dealing with these distinct “fitness-relevant” coordination problems over time has selected for contingent leadership prototypes to aid in the swift endorsement of appropriate context-specific leaders (see Spisak et al., 2011). Leadership increases the efficiency and effectiveness of collective action and taking too long to coordinate or following the wrong leader can severely hinder the fitness-enhancing value of a social group strategy (Van Vugt et al., 2008). The skills required to dominate competitors, for example, can be a hindrance when attempting to create and maintain cooperation between groups. In a contemporary context, this inability to correctly assign leadership may be one of the reasons why researchers find that approximately half of all mergers and acquisitions fail (Cartwright and Schoenberg, 2006). Some organizations may simply take a “one size fits all” approach to leadership and dominant agents maintain their hierarchical authority when more prosocial leadership should be allowed to emerge.

Research on shared leadership, where distributing leadership across a number of individuals can significantly enhance group performance (e.g., Carson et al., 2007), provides a clear connection between repetitive organizational challenges and evolved leader prototypes. Here we aim to understand how evolution may have shaped our implicit preferences for shared leadership. Specifically, we are investigating cognitive associations between the evolutionarily consistent coordination pressures mentioned above and contingent leader qualities which may have been selected for as part of human followership psychology. Such efforts advance our understanding of contingent decision-making which consequently helps to maximize the benefits of shared leadership (i.e., selecting the right leader for the situation as opposed to one size fits all).

To understand this cognitive process one must first consider how such contingencies are executed to produce leadership emergence. A prominent cue for this purpose is the human face, which provides a wealth of information about an individual, including information about character traits and genetic fitness (Bruce and Young, 2012). We more specifically know that individuals can assess leadership success of political candidates better than chance by mere exposure to their photograph (Todorov et al., 2005), and children as young as 5 years old can replicate this outcome (Antonakis and Dalgas, 2009). The latter sample of children (who are void of political experience) suggests that such judgments have less to do with social stereotypes of politicians and more to do with a deeper cognitive bias triggered by information embedded in the face.

The face stores a significant amount of useable data for context-specific leadership decision-making. Qualities such as facial femininity or perceived age can have a significant impact on who followers endorse as a leader in different situations because these visual signals can serve as a proxy for latent behavioral potential (e.g., Little et al., 2007). Estrogen levels, for example, are positively associated with both perceived facial femininity (Smith et al., 2006) as well as nurturing and affiliative behaviors (i.e., tending and befriending; Taylor et al., 2000) suggesting that the human face can serve as a reliable cue when selecting context-specific leaders (e.g., feminine face = tending and befriending = peace leader). Followers also seem to use a categorization approach with multiple levels of discrimination (see Spisak et al., 2012). Followers decide whether in the first-order a person looks like a leader in general and in the second-order relies on context-specific cues for decision-making (e.g., feminine face = peace leader).

A first-order facial cue that appears to generally (and positively) influence the perception of others is attractiveness—known as the “attractiveness halo” (see Moore et al., 2011). Included in this positive halo is leadership endorsement (Verhulst et al., 2010) and it is therefore important to accurately assess how this biasing process favoring attractive leaders operates. Employing a contingent categorization approach provides a useful framework for further clarification. The reason being is that attractiveness is associated with perceived facial health and perceived facial intelligence (see Zebrowitz and Rhodes, 2004) both of which have been argued to be important traits for leadership (e.g., Antonakis et al., 2009; Björklund et al., 2013). Thus, we can split apart the first-order attractiveness halo and search for context-specific second-order effects of health and intelligence, thereby expanding the boundary of understanding for both leadership categorization and context-specific cognitive prototyping.

This approach generates a number of relevant questions regarding implicit leadership processes. For instance, based on an implicit match between contextual requirements and distinct qualities associated with cues of intelligence and health, will leaders who look relatively more intelligent be favored in situations where experience or knowledge is more important and will group members be more likely to follow healthier-looking leaders in physically demanding circumstances? In addition, given that these cognitive contingencies would have developed over the course of human evolution, will they still hold in modern organizational settings? Signals of health are perhaps exceptionally important in dynamics which traditionally required a leader to exert an increased amount of physical energy such as during intergroup conflict. However, modern competition does *not* necessarily require physical action. That said, it appears that despite such discrepancies competitive environments in business still tend to select for individuals high in risk-taking and testosterone (Sapienza et al., 2008) indicating that the underlying contingency logic and associated leadership prototypes of these coordination challenges remain intact.

In the current paper we work to further our understanding of leadership by activating contemporary versions of the coordination problems described above (i.e., competition vs. cooperation and exploration vs. exploitation) and pairing these group challenges with faces of potential leaders where first-order attractiveness is controlled for and the subcomponents of health and intelligence are independently manipulated. It is clear that over the course of human evolution, the aggressive nature of *competition* had a significant physical component (e.g., Keeley, 1996) and we therefore expect followers to contingently prefer healthier-looking leaders over intelligent-looking leaders. Conversely, maintaining prosocial *cooperation* between groups through tending and befriending strategies such as trust building and empathy is mentally taxing—demanding *both* cognitive and emotional processing (Penner et al., 2005). Thus, in cooperative between-group situations, it is expected that followers will contingently prefer intelligent-looking leaders over healthier-looking leaders. As for exploration vs. exploitation, it is first important to note that the cognitive adaptations driving our exploratory vs. exploitative decision-making are relatively understudied (Cohen et al., 2007) and it is therefore important to approach cautiously. In groups, *exploration* of new resource opportunities traditionally required relatively increased physical output (MacArthur and Pianka, 1966) and as a result we predict that healthier-looking leaders will be preferred. However, ensuring a group stabilizes and maintains consistent exploitation of an established resource requires the utilization of existing knowledge and past experience (e.g., crystalized intelligence; Cattell, 1987) relative to physical ability and we expect intelligent-looking leaders to contingently match this situation. Finally, followers likely prefer leaders to be *both* healthy and intelligent, but by separating these subcomponents one can better understand what is driving the attractiveness halo in leadership decisions and more accurately model its impact on leadership emergence in diverse situations.

## Materials and methods

### Participants

One hundred and 48 participants (79 males, 69 females, *M*_age_ = 33.1, *SD* = 11.8) completed an online experiment for financial compensation. The experiment was made using Qualtrics and distributed to Mturk users with Crowdflower. The original dataset consisted of 191 participants. We deleted participants who did not complete the experiment, participants who failed a simple reading test (“This question tests whether you are reading the questions and answers. Please answer 3”), and participants who failed more than 1 out of 4 manipulation checks (manipulation checks tested whether participants could identify which scenario they had just answered questions on).

### Procedure

The procedure for the experiment consisted of three separate tasks. First, the facial stimuli used for testing were created. Second, business scenarios based on the coordination problems mentioned above were developed. These materials were then combined to run the experiment. Finally, the created faces were rated by two samples on perceived health, intelligence, masculinity, and attractiveness.

#### Health and intelligence face morph materials

Stimuli were created using Psychomorph (Tiddeman et al., 2001), custom built software for the graphical manipulation of facial photographs. First, we created four base identities, each by combining three individual faces of undergraduate white men who were all clean shaven and had no glasses or visible jewelry. We combined faces such that both perceived intelligence and attractiveness were matched, based on previous ratings of the individual faces (*N* = 14 raters). This procedure ensured that differences between stimuli in perceived health and intelligence were driven exclusively by our transforms and not by idiosyncratic differences between stimuli.

Next, each identity was transformed in apparent intelligence. To this end, high and low apparent intelligence prototypes were created as described in Moore et al. (2011). Briefly, these prototypes were created by regressing ratings of attractiveness, masculinity, health, and perceived age against ratings of perceived intelligence. The faces with the largest positive and negative residuals (i.e., those who were rated as looking much more or less intelligent than predicted by their age, attractiveness, masculinity, and health) were “averaged” using Psychomorph software to create composite high and low perceived intelligence faces. Subsequently, each base identity was transformed in face shape by ±50% of the linear shape difference between the high intelligence and low intelligence prototype, yielding 2 versions of each identity: one high intelligence version and one low intelligence version. Moderate manipulations of the two versions (i.e., high and low intelligence) were also created by reducing the transform to ±25%.

Third, we next transformed both the high and low intelligence versions of each identity to be high or low on apparent health. To this end, we manipulated the skin areas of each face to appear lower or higher in carotenoid-associated skin coloration, observed following increased fruit and vegetable consumption (see Whitehead et al., 2012) and reliably perceived as healthy looking (e.g., Stephen et al., 2009). To simulate an increase in health appearance we added 4.35 units of yellowness (b^*^ in the CIELab color space, see Stephen et al., 2009 for details), subtracted 1.1 units of lightness (L^*^) and added 1.4 units of redness (a^*^) to all faces. To simulate a decrease in healthy appearance, the reverse manipulation was performed. The levels of positive transform were derived from a previous study, which indicated that on average, this amount of color change was applied to Caucasian faces to make them appear most healthy (see Lefevre et al., 2013). In addition, we created a moderate health transform version, so as to ensure that the transform would be more closely aligned in magnitude with the two levels of the intelligence transform. To this end, we halved the amount of color added and subtracted, in other words, we added 2.18 units of b^*^, subtracted 0.55 units of L^*^ and added 0.7 units of a^*^ to each face to create the medium level healthy face. The medium level unhealthy face was created by reversing this manipulation.

**Figure 1:**
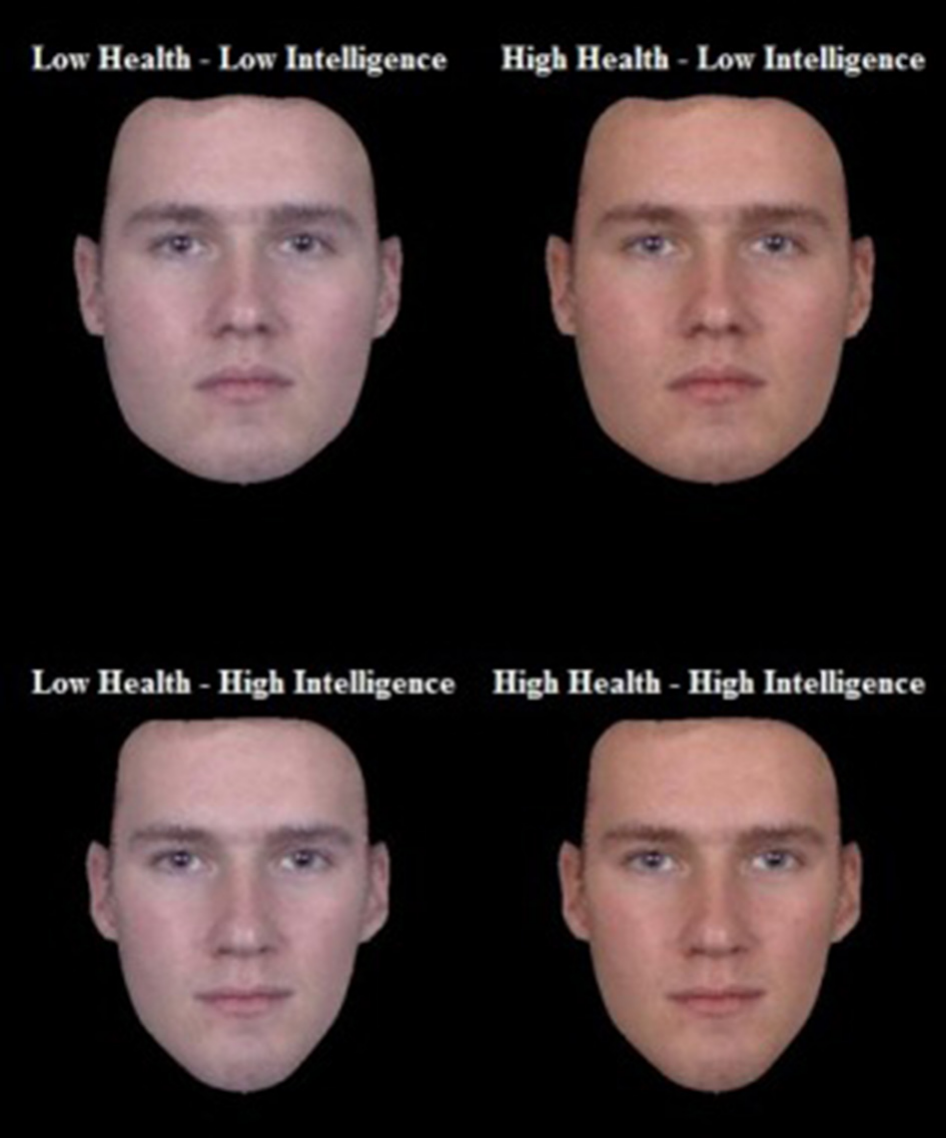
**Example of the four face types created by independently manipulated high and low signals of health and intelligence**.

To sum up the procedure, facial shape was first adjusted to alter perceptions of intelligence, creating high intelligence (Hi) and low intelligence (Li) versions of the base faces. Next, the coloration of Hi and Li facial images where manipulated to create high health (Hh) and low health (Lh) version. This process yielded four face types (i.e., HiHh, LiLh, HiLh, and LiHh; Figure 1). To examine possible thresholds for perceiving difference between health and intelligence we also created medium and strong versions of the four face types by adjusting the transform percentages. Images were then cropped to the outer boundaries of the face. The transforms thus created a total of 32 faces. Four different male composite base faces, of which each had four health/intelligence versions (HiHh, LiLh, HiLh, and LiHh), all of which had a 25% and a 50% transform version (4 * 4 * 2 = 32).

#### Experimental procedure

The next step was to pair the face types with business scenarios based on the four coordination dynamics identified in the introduction (i.e., competition, cooperation, exploration, and exploitation; see Supplemental Materials for the scenarios). The objective was to investigate which subcomponent of attractiveness (i.e., health or intelligence) would be preferred in each coordination dynamic. To accomplish this, each scenario was presented one at a time with one male base face in all possible paired combinations of the four face types presented below, six combinations in total (e.g., HiHh vs. LiLh, HiLh vs. LiHh). We counterbalanced which male base face was paired with which scenario, and also counterbalanced the order in which the different scenarios and different male base faces were presented. Per scenario, participants thus chose their preferred leader out of two faces (both coming from the same base face but transformed differently) six times. Each participants made 24 (6 combinations ^*^ 4 scenarios) leadership decisions, either with a transform level of 25%, or a transform level of 50% (transform level varied between subjects).

The scenario appeared at the top of the screen and the participant was presented with the first pair of faces and asked to vote for the face they would prefer as a leader for the depicted scenario (i.e., forced-choice pairing). Once a decision was made, the next face pair would appear below the scenario and the participant would make another leader choice. This procedure continued until all six paired face combinations had been displayed with the scenario. Then the scenario would switch and the procedure would repeat until a decision for all face combinations were made for all four scenarios. Scenarios, face pairings, and side of the monitor where the face appeared were randomized to control for order effects. Scenario and assigned faces were randomized to control for idiosyncratic effects of any one particular face paired with any one scenario. Following the leadership selection task, participants explicitly rated the faces on perceived health, intelligence, attractiveness, and masculinity (e.g., “This person looks attractive,” 1 = *strongly disagree*, 10 = *strongly agree*). The experimental design was approved by the ethics committee at the VU University Amsterdam. Before the experiment informed consent was obtained and following the tasks participants were thanked and debriefed.

## Results

### Ratings of health, intelligence, and attractiveness

In order to get some insight into how the faces were perceived, we had all faces rated on health, intelligence, masculinity, and attractiveness, by two samples. All ratings were performed on a scale ranging from 1 (*strongly disagree*) to 10 (*strongly agree*). The first sample (*N* = 105, 69 female/36 male, *M*_age_ = 36.46, *SD*_age_ = 12.69) collected via MTurk performed the face ratings separately before we conducted the actual main study, and thus did not complete any other parts of the experiment (i.e., they did not choose leaders for different scenarios). This first sample originally consisted of 118 participants—those who failed a reading test or a manipulation check (“What gender were faces in this experiment?”) were deleted from the dataset. The second sample consisted of the 148 participants of the actual experiment (who performed the ratings after they had completed the leadership selection task in all four scenarios).

Tables 1, 2 summarize the mean ratings of health, intelligence, attractiveness, and masculinity per manipulation. The ratings in the high health columns of Tables 1, 2 are the average ratings of perceived health of the two face types with high health transforms (i.e., Hi**Hh** and Li**Hh**), while the ratings in the low health columns are the average ratings of perceived health of the two face types with low health transforms (i.e., Li**Lh** and Hi**Lh**). The same goes for the high and low intelligence columns—under high intelligence are the average ratings from the two transforms of the high intelligence faces (i.e., **Hi**Hh and **Hi**Lh), and under low intelligence are the average ratings from the two transforms of the low intelligent faces (i.e., **Li**Lh and **Li**Hi). These scores are the average of the 25% and 50% transforms—if the main analysis shows that transform strength affects how our manipulations influence leader selection, we planned to revisit the ratings separately for the 25% and the 50% transforms. The ratings show that the high health faces are indeed perceived healthier than the low health faces, and that the high intelligence faces are seen as higher in intelligence than the low intelligence faces, as the manipulations intended. However, other cues are also affected by the health and intelligence manipulations. For instance, participants rate the high health and high intelligence faces higher on attractiveness than the low health and low intelligence faces. Additionally, the high health faces are perceived as more masculine than the low health faces, whereas intelligence has the opposite effect—the low intelligent faces are seen as more masculine than the high intelligent faces. Most effects of the health and intelligence manipulations on ratings are of small to medium size (as denoted by Cohen's D), with a notable exception of a larger effect of the health manipulation on perceived health in the second sample. A preference for a high health face over a low health face, and a preference for a high intelligence face over a low intelligence face, may thus be explained by a combination of subjective perceptions of health, intelligence, masculinity, and attractiveness.

**Table 1:** **Sample 1 (*N* = 105) Ratings of high health vs. low health faces, and ratings of high intelligence vs. low intelligence faces—Means, SDs, *t*-tests, and Cohen's Ds**.

	**High Health**	**Low Health**	**Difference**
Health	6.56 (1.77)	6.28 (1.81)	*t* = 3.81, *p* < 0.001^*^, *d* = 0.37
Intelligence	6.09 (1.57)	5.88 (1.61)	*t* = 4.08, *p* < 0.001^*^, *d* = 0.40
Attractiveness	5.34 (1.84)	5.10 (1.91)	*t* = 3.53, *p* = 0.001^*^, *d* = 0.34
Masculinity	6.60 (1.98)	6.41 (2.13)	*t* = 2.91, *p* = 0.004^*^, *d* = 0.28
	**High Intelligence**	**Low Intelligence**	**Difference**
Health	6.54 (1.72)	6.30 (1.88)	*t* = 2.89, *p* = 0.005^*^, *d* = 0.45
Intelligence	6.17 (1.57)	5.80 (1.66)	*t* = 4.62, *p* < 0.001^*^, *d* = 0.28
Attractiveness	5.36 (1.86)	5.07 (1.92)	*t* = 3.51, *p* = 0.001^*^, *d* = 0.34
Masculinity	6.41 (2.06)	6.60 (2.07)	*t* = −2.35, *p* =0.020, *d* = −0.23

* *p remains <0.05 after adjusting for multiple comparisons (Bonferroni correction)*.

**Table 2:** **Sample 2 (*N* = 148) Ratings of high health vs. low health faces, and ratings of high intelligence vs. low intelligence faces—Means, SDs, *t*-tests, and Cohen's Ds**.

	**High Health**	**Low Health**	**Difference**
Health	7.37 (1.59)	6.49 (2.01)	*t* = 8.17, *p* < 0.001^*^, *d* = 0.67
Intelligence	7.00 (1.65)	6.81 (1.77)	*t* = 2.47, *p* = 0.015, *d* = 0.20
Attractiveness	6.14 (2.01)	5.63 (2.15)	*t* = 5.95, *p* < 0.001^*^, *d* = 0.30
Masculinity	7.34 (1.76)	7.03 (1.95)	*t* = 3.84, *p* < 0.001^*^, *d* = 0.32
	**High Intelligence**	**Low Intelligence**	**Difference**
Health	7.15 (1.75)	6.70 (1.99)	*t* = 3.34, *p* = 0.001^*^, *d* = 0.27
Intelligence	7.09 (1.80)	6.71 (1.79)	*t* = 3.13, *p* = 0.002^*^, *d* = 0.26
Attractiveness	6.15 (2.18)	5.63 (2.20)	*t* = 3.69, *p* < 0.001^*^, *d* = 0.30
Masculinity	6.98 (1.97)	7.39 (1.96)	*t* = −3.11, *p* = 0.002^*^, *d* = −0.26

* *p remains <0.05 after adjusting for multiple comparisons (Bonferroni correction)*.

It is also interesting to consider the different perceptions of the two opposed-combination faces, i.e., the low intelligence but high health face (LiHh), and the high intelligence but low health face (HiLh). First, the high health but low intelligence face is perceived as more masculine in both samples [sample 1 − *t*_(104)_ = 3.60, *p* < 0.001, *d* = 0.35, sample 2 − *t*_(147)_ = 4.91, *p* < 0.001, *d* = 0.40]. Second, while the low health but high intelligence face is rated more intelligent than the low intelligence but high health face in both samples [sample 1 − *t*_(104)_ = −2.03, *p* = 0.045, *d* = −0.20, sample 2 − *t*_(147)_ = −1.22, *p* = 0.225, *d* = −0.10], the difference is only significant in the first sample. Third, the high health but low intelligence face is rated more healthy in the second sample, but there is no difference in ratings between the two face types concerning health ratings in the first sample [sample 1 − *t*_(104)_ = 0.35, *p* = 0.730, *d* = 0.03, sample 2 − *t*_(146)_ = 2.42, *p* = 0.017, *d* = 0.20]. Finally, there is no difference in perceived attractiveness between the high health but low intelligence face, and the low health but high intelligence face (sample 1 and 2-*t* < 1, *p* = ns). A preference for one of these opposed-combination face types over the other will thus not be driven by a difference in attractiveness, but may be guided by perceptions of health, intelligence, and masculinity.

### Predicting leader selection by health and intelligence

To analyze the data we utilized a version of the Bradley-Terry Model which uses a log-linear approach to account for the dependence between multiple paired comparisons from a given set of objects (Dittrich et al., 2002). This statistical technique allowed us to analyze voting preferences for each face-type separately (i.e., HiHh, LiLh, HiLh, and LiHh) while accounting for the interdependency of multiple paired comparisons within participants. Subsequently, we were able generate a 2 × 2 design to investigate main effects of intelligence (high vs. low) and health (high vs. low). We combined the 25% and 50% transforms for the analyses, with the plan to revisit the two transform levels separately should the analysis show that transform level affects results.

On average (taken across all 4 scenarios), health had a significant positive effect on leader selection [Wald χ^2^_(*df* = 1)_ = 136.30, *p* < 0.001], as did intelligence [Wald χ^2^_(*df* = 1)_ = 26.51, *p* < 0.001]. There were no significant main effects of participant gender [Wald χ^2^_(*df* = 1)_ = 2.587, *p* = 0.108], scenario [Wald χ^2^_(*df* = 1)_ = 0.005, *p* > 0.999], or manipulation strength [Wald χ^2^_(*df* = 1)_ = 0.015, *p* = 0.901] on leader selection.

Health was a significant predictor of leadership ratings in all four scenarios; in cooperation [Wald χ^2^_(*df* = 1)_ = 22.01, *p* < 0.001], competition [Wald χ^2^_(*df* = 1)_ = 38.00, *p* < 0.001], exploration [Wald χ^2^_(*df* = 1)_ = 32.42, *p* < 0.001], and exploitation [Wald χ^2^_(*df* = 1)_ = 36.10, *p* < 0.001). On the other hand, intelligence led to an increase in leader selection in the exploration condition [Wald χ^2^_(*df* = 1)_ = 24.06, *p* < 0.001), along with an increase in the cooperation condition [Wald χ^2^_(*df* = 1)_ = 19.24, *p* < 0.001), but had no positive effect on leader selection in the competition [Wald χ^2^_(*df* = 1)_ = 0.18, *p* = 0.674] or exploitation conditions [Wald χ^2^_(*df* = 1)_ = 0.73, *p* = 0.434]. Overall, health thus had a positive effect on leader selection in all four scenarios, while intelligence only showed this effect in the exploration and cooperation conditions.

Because we summed across the medium and strong manipulation in the above analyses, we wanted to make sure that there were no interactions of manipulation strength with intelligence or health on leader selection; a significant interaction would imply we need to look at the medium and strong manipulation conditions separately. We performed another analysis across all 4 scenarios together, adding the interaction terms (manipulation strength ^*^ intelligence, and manipulation strength ^*^ health) to the model. There was no significant interaction between health and manipulation strength on leader selection [Wald χ^2^_(*df* = 1)_ = 0.019, *p* = 0.890], and no interaction between intelligence and manipulation strength on leader selection [Wald χ^2^_(*df* = 1)_ = 1.089, *p* = 0.297].

#### Health vs. intelligence

We then wanted to see whether one cue had a stronger effect on decision making than the other. Health was the stronger predictor for the exploration scenario [*t*_(148)_ = 2.241, *p* = 0.013], the exploitation scenario [*t*_(148)_ = 4.336, *p* < 0.001), and the competitive scenario [*t*_(148)_ = 5.099, *p* < 0.001]. There was no significant difference in predictor strength between health and intelligence in the cooperation scenario. [*t*_(148)_ = 1.306, *p* = 0.192). Finally, health had an overall stronger effect on leadership ratings than intelligence [*t*_(148)_ = 7.027, *p* < 0.001].

#### Comparing predictors across scenarios

We next tested whether health and intelligence had a stronger effect in one scenario relative to another. We were interested in two particular comparisons: the effects of health and intelligence in the competitive vs. the cooperative scenario—tested by combining the data of these two scenarios and testing the interaction between health/intelligence and scenario on leader selection—and the effects of health and intelligence in the exploration vs. exploitation scenario—again, tested by combining the data of these two other scenarios and testing the interaction between health/intelligence and scenario on leader selection. As expected, intelligence was a stronger predictor in the cooperation scenario than in the competition scenario [Wald χ^2^_(*df* = 1)_ = 18.796, *p* < 0.001). However, contrary to expectations, intelligence was a stronger predictor in the exploration scenario than in the exploitation scenario [Wald χ^2^_(*df* = 1)_ = 12.154, *p* < 0.001]. Results also showed that health was an equally strong predictor in the cooperation vs. the competition scenario [Wald χ^2^_(*df* = 1)_ = 1.213, *p* = 0.271], and also did not differ in strength in the exploration vs. the exploitation scenario [Wald χ^2^_(*df* = 1)_ = 0.382, *p* = 0.537].

Table 3 gives an overview of how often participants chose a high health face over a low health face, and how often participants chose a high intelligent face over a low intelligent face, across all trials. In line with the main results, these percentages show that while there are some scenarios where high intelligence faces are only favored slightly above chance (i.e., competition and exploitation), the high health faces are always preferred well above chance.

**Table 3:** **Percentages of choices for high health faces over low health faces and choices for high intelligence faces over low intelligence faces**.

	**Overall (%)**	**Competition (%)**	**Cooperation (%)**	**Exploration (%)**	**Exploitation (%)**
High Health wins from Low Health	69.4	68.7	67.3	71.9	69.8
High Intelligence wins from Low Intelligence	63.8	53.7	70.1	73.1	58.0

## Discussion

To summarize, health and intelligence both influenced leader selection, but the health cue (facial color) was clearly more influential than the intelligence cue (facial structure) in our scenarios. Health was an influential cue across all scenarios, while intelligence only had an effect in half of the presented scenarios. Overall, health was a significantly stronger predictor of leader selection than intelligence, except for in the cooperation context, where intelligence and health were predictors of similar strength. Our results indicate a stronger general preference for health vs. intelligence when selecting leaders across context.

As for our hypotheses, we found mixed support. In leader selection, cues of intelligence, as expected, were preferred more often in cooperation vs. competition whereas perceived health was significantly favored across all four coordination problems. As for exploration vs. exploitation, to date, it has had limited research attention in the behavioral and brain sciences (Cohen et al., 2007) and future research may provide insights into whether our initial predictions regarding prototypical contingencies are accurate. Overall, our findings suggest that although intelligence may be important for leadership in certain circumstances, health (represented by facial coloration based on increased carotenoid pigmentation) appears to dominate decision making in all contexts of leadership. In terms of categorization, this means that leaders relatively high in perceived intelligence have a second-order, contextually-bound advantage—such as in times requiring between-group cooperation—whereas healthier-looking leaders perhaps have a context-general, first-order advantage across a diverse landscape of leadership situations. This aligns with recent work suggesting that the activation of “disease concerns” in the environment exacerbates the voting tendency to prefer attractive political candidates. Attractiveness is in part driven by cues to health and healthy leaders are likely to be exceptionally important when disease threatens the viability of the group (White et al., 2013). Adding to this, our data indicates that with or without specific pathogen threats health is generally an important factor when selecting leaders.

While the facial health and intelligence manipulations predictably affected participants' ratings of perceived health and intelligence, it is important to note that the manipulations also affected perceptions on other dimensions, such as attractiveness and masculinity. It is apparent from our results that our transforms did change perceptions of attractiveness. However, this was the objective of our research (i.e., to assess which specific dimensions of attractiveness affect leadership perception). We also note in our results that perceptions of attractiveness did *not* significantly differ between high intelligence but low health and low intelligence but high health faces (i.e., HiLh vs. LiHh). Furthermore, while our transforms did also affect perceived masculinity this effect likely does not entirely explain our main effects of health and intelligence on leadership choice for the following reason: Increased health and increased intelligence positively affected leadership perceptions; however, masculinity ratings increased in the high health transform but *decreased* in the high intelligence transform. Also, while we can conclude from our data that increased facial carotenoid pigmentation—a marker for physical health—increases whether someone is preferred as a leader, we have to be more careful with drawing strong conclusions about how facial intelligence affects leader preference. Whereas facial coloration is an objective cue for health, our intelligence manipulation is based on subjective perceptions of low and high intelligence. This subjective intelligence transform may actually be a reflection of other objective cues which were more salient to the participants such as, in this case, facial masculinity (i.e., our low intelligence faces may actually have more masculine features than the high intelligence faces). Thus a better understanding of the relationship between facial masculinity and perceived intelligence is an important next step for drawing a sound conclusion about facial intelligence and leadership preferences.

The ratings of faces high in one positive cue but low in another positive cue—i.e., HiLh vs. LiHh—have additional implications. The ratings from two separate samples suggest that picking up on a high health cue (facial coloration) seems more difficult when the facial structure is characteristic of low intelligence, and vice versa, picking up on cues for high intelligence seems more difficult when there is a clear competing cue for low health. However, when a face has low intelligence combined with high health facial coloration, perceptions of masculinity are particularly enhanced. These results demonstrate how a facial cue can have different effects when combined with other cues, and that novel perceptions may arise from a specific combination of cues—an interesting avenue for future research.

Like much previous research, our results demonstrate that morphological cues can guide decision making when it comes to leadership. From an organizational science perspective, this means that, for instance, leadership succession planning, external hiring of managers and executives, and general willingness to follow a leader are likely biased by a variety of such cues. We must then account for these biases and work with or around such cognitive shortcuts. As an example, a relatively healthy-looking leader may have a better chance of gaining sufficient levels of followership investment to initiate change. On the other hand, a potential leader who looks relatively less healthy may be overlooked even if they are better suited for the job—the difference between emergence and effectiveness.

There are also a number of limitations to the current study that deserves mentioning. First, leadership selection for the exploration-exploitation dilemma needs further development. Continued effort is necessary to identify and match the contingent leadership traits associated with both exploration and exploitation. Second, intelligence is a somewhat broad concept. The difference between fluid and crystallized intelligence (i.e., the ability to develop novel solutions to novel problems vs. the ability to use acquired knowledge, skills, and experience; e.g., Cattell, 1987) are perhaps best suited for exploration vs. exploitation, respectively. Future work should investigate perceptual differences between these types of intelligence. Existing research on the developmental differences between fluid vs. crystallized intelligence (e.g., Horn and Cattell, 1967) suggests that facial cues of age may serve as a proxy when perceptually attributing these two types of intelligence (i.e., young = fluid and old = crystallized) and, as a consequence, this could create a contingent match between young exploration leaders and old exploitation leaders. Further use of the contingent categorization approach can provide a framework for constructing a network of first- and second-order cues and how they shift in importance across context. Finally, the scenarios used in this study, designed to represent situations characterized by cooperation, competition, exploration, or exploitation, had some specific details which may have affected decision making. For instance, the between group competition scenario may have elicited a particularly individual-level focus (the situation concerned everyone, but “especially you”), while the between group cooperation scenario may have also enhanced stronger feelings of group identification (the focus here is on “your colleagues,” and not on “especially you”) due to wording of the scenarios. Replication of our main results with different scenarios is necessary to test how robust these results are.

A modern version of implicit leadership categorization that contingently considers the dynamics of fitness-relevant situations is an effective approach for understanding why certain leaders emerge when they do. Our results demonstrate that when one attempts to split apart perceived facial attractiveness into second-order categories they immediately discover a general preference for health, characterized by facial coloration, when selecting leaders. Thus health is a first-order categorization variable that initially biases us to perceive a potential candidate as a leader in general or *not*. This adds an attractive twist to research on beauty and its impact on followers.

### Conflict of interest statement

The authors declare that the research was conducted in the absence of any commercial or financial relationships that could be construed as a potential conflict of interest.
