# Supplementary material for: A face for all seasons: Searching for context-specific leadership traits and discovering a general preference for perceived health
Source: Front Hum Neurosci. 2014 Nov 5;8:792. doi: 10.3389/fnhum.2014.00792 (PMC4221639; doi:10.3389/fnhum.2014.00792)
Supplement: Supplementary file 1 [file DataSheet1.DOCX]

***Supplementary Material***

**A face for all seasons: Searching for context-specific leadership traits and discovering a general preference for perceived health**

**Brian R. Spisak^1,*^, Nancy M. Blaker^2^, Carmen E. Lefevre^3^, Fhionna R. Moore^4^, & Kleis F. B. Krebbers^1^**

^1^Department of Management and Organization, VU University Amsterdam, Amsterdam, NL.

^2^Department of Social and Organizational Psychology, VU University Amsterdam, Amsterdam, NL

^3^Centre for Decision Research, Leeds University Business School, Leeds, UK

^4^School of Psychology, University of Dundee, Dundee, UK.

**^*^Correspondence:** Dr. Brian R. Spisak, VU University Amsterdam, Department of Management and Organization, De Boelelaan 1105, 1081 HV, Amsterdam, The Netherlands.

b.r.spisak@vu.nl

**1. Supplemental scenarios**

**1.1. Competition – between groups**

Imagine your company, called TAM Inc., is battling with its biggest competitor, ROB Inc., for market share. It has been aggressive and costly competition with no side willing to back down. Recently, ROB Inc. has increased their marketing activities and intensified the battle for customers and market shares. This has made everyone, especially you, concerned about the future of your company. You and your colleagues are determined to establish dominance over ROB Inc. in order to increase maintain share, and to protect the employees of TAM Inc. Currently, your company is searching for a new CEO and multiple board members are in the race to become the next CEO. You will see multiple pairings of the board members photographs. For each face pairing select the one you think is best for successfully leading competition against ROB Inc.

**1.2. Cooperation – between groups**

Imagine your company, called TAM Inc., has a cooperative relationship with the company ROB Inc. This alliance, however, has become strained due to conflicting policies. Both sides threaten to restrict cooperation and stop collaborating. Also, it has been rumored that ROB Inc. will terminate the alliance within the next six months and is likely to collaborate with your greatest competitor. If that happens, a large decline of market share is almost certain. Your fellow colleagues at TAM Inc. feel that competition against ROB Inc. is something that should be avoided at all cost. Instead, stakeholders want to restore and increase cooperation with ROB Inc. through respectful and professional negotiations. Currently, your company is searching for a new CEO and multiple board members are in the race to become the next CEO. You will see multiple pairings of the board members photographs. For each face pairing select the one you think is best for successfully leading cooperation between TAM Inc. and ROB Inc.

**1.3. Exploration**

Imagine your company, called TAM Inc., is in a time of change and exploration. TAM Inc. has committed itself to exploring renewable resources such as solar and wind energy for its energy demand. It is important for the sustained success of TAM Inc. to ensure that this exploratory change happens. The employees of TAM Inc. all agree that exploring alternative energy is of the utmost importance. Currently, your company is searching for a new CEO and multiple board members are in the race to become the next CEO. You will see multiple pairings of the board members photographs. For each face pairing select the one you think is best for leading change to renewable resources such as wind and solar energy.

**1.4. Exploitation**

Imagine your company, called TAM Inc., is currently in need of stable exploitation of nonrenewable resources. TAM Inc. depends on coal and oil. It is important for the sustained success of TAM Inc. that these nonrenewable resources are conserved and not over-used. The employees of TAM Inc. all agree that stable exploitation of nonrenewable resources is of the utmost importance. Currently, your company is searching for a new CEO and multiple board members are in the race to become the next CEO. You will see multiple pairings of the board members photographs. For each face pairing select the one you think is best for leading stability of nonrenewable resources such as coal and oil.
